# Differential vulnerability of neuronal subpopulations of the subiculum in a mouse model for mesial temporal lobe epilepsy

**DOI:** 10.3389/fncel.2023.1142507

**Published:** 2023-03-29

**Authors:** Julia Franz, Nicole Barheier, Henrike Wilms, Susanne Tulke, Carola A. Haas, Ute Häussler

**Affiliations:** ^1^Experimental Epilepsy Research, Department of Neurosurgery, Faculty of Medicine, Medical Center-University of Freiburg, Freiburg, Germany; ^2^Faculty of Biology, University of Freiburg, Freiburg, Germany; ^3^Center for Basics in NeuroModulation, Faculty of Medicine, University of Freiburg, Freiburg, Germany; ^4^BrainLinks-BrainTools, University of Freiburg, Freiburg, Germany

**Keywords:** parvalbumin, neuropeptide Y, calretinin, FluoroJade C, intrahippocampal kainate, dorso-ventral axis, epileptic activity

## Abstract

Selective loss of inhibitory interneurons (INs) that promotes a shift toward an excitatory predominance may have a critical impact on the generation of epileptic activity. While research on mesial temporal lobe epilepsy (MTLE) has mostly focused on hippocampal changes, including IN loss, the subiculum as the major output region of the hippocampal formation has received less attention. The subiculum has been shown to occupy a key position in the epileptic network, but data on cellular alterations are controversial. Using the intrahippocampal kainate (KA) mouse model for MTLE, which recapitulates main features of human MTLE such as unilateral hippocampal sclerosis and granule cell dispersion, we identified cell loss in the subiculum and quantified changes in specific IN subpopulations along its dorso-ventral axis. We performed intrahippocampal recordings, FluoroJade C-staining for degenerating neurons shortly after *status epilepticus* (SE), fluorescence *in situ* hybridization for glutamic acid decarboxylase (*Gad) 67* mRNA and immunohistochemistry for neuronal nuclei (NeuN), parvalbumin (PV), calretinin (CR) and neuropeptide Y (NPY) at 21 days after KA. We observed remarkable cell loss in the ipsilateral subiculum shortly after SE, reflected in lowered density of NeuN+ cells in the chronic stage when epileptic activity occurred in the subiculum concomitantly with the hippocampus. In addition, we show a position-dependent reduction of *Gad67*-expressing INs by ∼50% (along the dorso-ventral as well as transverse axis of the subiculum). This particularly affected the PV- and to a lesser extent CR-expressing INs. The density of NPY-positive neurons was increased, but the double-labeling for *Gad67* mRNA expression revealed that an upregulation or *de novo* expression of NPY in non-GABAergic cells with a concomitant reduction of NPY-positive INs underlies this observation. Our data suggest a position- and cell type-specific vulnerability of subicular INs in MTLE, which might contribute to hyperexcitability of the subiculum, reflected in epileptic activity.

## 1. Introduction

Mesial temporal lobe epilepsy (MTLE) is among the most frequent forms of pharmacoresistant epilepsies. To elucidate the underlying pathophysiological mechanisms, the hippocampus as site of origin of focal seizures has been intensely investigated. This revealed histological alterations including hippocampal sclerosis, granule cell dispersion and mossy fiber sprouting as hallmarks of MTLE in patients and animal models (Sutula et al., 1989; Houser, 1990; Sloviter, 1994; Suzuki et al., 1995; Haas et al., 2002; Thom et al., 2002; Blümcke et al., 2013; Häussler et al., 2016). In contrast, the subiculum received less attention, despite playing a pivotal role in the network as a major output region of the hippocampal formation.

With its large proportion of bursting cells (Taube, 1993; Greene and Totterdell, 1997) and a network that facilitates synchronization of neuronal activity (Behr and Heinemann, 1996; Fiske et al., 2020), the subiculum is an ideal candidate region for the generation of epileptic activity. Moreover, the subiculum becomes subject of considerable deafferentation due to extensive cell loss in the CA1 region in patients (Blümcke et al., 2013) and animal models (Suzuki et al., 1995; Janz et al., 2017a) which might induce network imbalance.

Indeed, using acute brain slices from MTLE patients undergoing resective surgery, it has been proposed that the subiculum is the origin of spontaneous inter-ictal activity (Cohen et al., 2002; Wozny et al., 2005), which was confirmed by *in vivo* recordings of patients with pharmacoresistant MTLE (Fabó et al., 2008). Interestingly, the highest probability for generation of paroxysmal high frequency oscillations (HFOs) switches from the hippocampus to the subiculum with increasing grades of hippocampal sclerosis (Tóth et al., 2021). On the histological level, pathological changes in the subiculum are less evident than those seen the sclerotic hippocampus: Overall neuronal numbers are only mildly affected in patient tissue (Arellano et al., 2004; Andrioli et al., 2007), but data on parvalbumin (PV)-expressing interneurons (INs) reach from no loss (Arellano et al., 2004) to more than 50% loss (Andrioli et al., 2007). Functionally, epileptic activity in the subiculum has been suggested to arise from impaired inhibition (Cohen et al., 2002).

Studies in animal models for MTLE have shown decreased overall neuronal density (Knopp et al., 2005; Drexel et al., 2012b) and loss of several IN populations [PV, calretinin (CR), somatostatin (SOM), and others; (He et al., 2010; Drexel et al., 2011, 2012a)]. However, the induction of MTLE by the systemic injection of a convulsant drug causes damage to the hippocampus and subiculum of both hemispheres and does not exactly reproduce the large patient cohort with unilateral hippocampal sclerosis (Blümcke et al., 2013).

To investigate changes in the subiculum of both hemispheres under conditions of severe focal, unilateral hippocampal sclerosis, we used the intrahippocampal kainate (ihKA) mouse model which reproduces the human characteristics particularly well (Suzuki et al., 1995; Depaulis and Hamelin, 2015). Our previous studies revealed that despite the focal KA injection, epileptic activity propagates to different subregions of the hippocampus proper, along large parts of the dorso-ventral (DV) axis, and to the contralateral hippocampus and the entorhinal cortex with different intensity (Froriep et al., 2012; Häussler et al., 2012; Janz et al., 2017b; Kilias et al., 2018, 2023; Tulke et al., 2019). It remains, however, unclear whether epileptic activity also affects the subiculum and whether this is associated with histological changes in this animal model.

Here, we show that not only generalized seizures but also subclinical epileptic activity affects the subiculum and is associated with neuronal loss, in particular loss of different IN populations to type- and position-specific degrees, depending on the hemisphere, the DV level and the position along the transverse axis of the subiculum.

## 2. Materials and methods

### 2.1. Animals

Experiments were performed in adult (>8 weeks) male negative littermates of transgenic mouse lines bred on C57Bl/6 background [B6(SJL)-Tg(Amigo2-icre/ERT2)1Ehs, C57BL/6-Tg(Thy1-eGFP)-M-Line, C57BL/6-Tg(Rbp4-cre)KL100Gsat or C57BL/6-Tg(Rbp4-cre)KL100Gsat x Ai32(RCL-ChR2)(H134R)/EYFP; CEMT Freiburg]. All lines have been shown to develop the full pattern of histological and electrophysiological characteristics of the ihKA model in a comparable manner in our earlier studies (Paschen et al., 2020; Kilias et al., 2023, Paschen/Kleis personal communication, own observations). Mice were housed under standard conditions [12 h-light/dark, room temperature (RT), food and water *ad libitum*]. Experiments were carried out following the guidelines of the European Community’s Council Directive of 22 September 2010 (2010/63/EU), approved by the regional council (Regierungspräsidium Freiburg). In total, *n* = 36 mice were used (*n*_NaCl_ = 14, *n*_KA_ = 22).

### 2.2. Surgery and *in vivo* recordings

Surgery was performed under deep anesthesia [ketamine hydrochloride 100 mg/kg body weight, xylazine 5 mg/kg, atropine 0.1 mg/kg, intraperitoneal (i.p.) injection] with analgesic treatment [buprenorphine hydrochloride 0.1 mg/kg, carprofen 5 mg/kg, subcutaneous (s.c.) injection; lidocaine 2 mg/kg, s.c. to scalp]. Mice were mounted in a stereotaxic frame and injections of 50 nL KA (20 mM in 0.9% NaCl, Tocris, Bristol, UK) or 0.9% NaCl into the right dorsal hippocampus were performed using a Nanoject III (Drummond Scientific Company, Broomall, PA, USA) at coordinates anteroposterior (AP) = −2.0 mm, mediolateral (ML) = −1.4 mm and DV = −1.8 mm from bregma. In KA-injected mice, behavioral *status epilepticus* (SE) with head nodding, convulsions, rotations and immobility was verified as previously described (Tulke et al., 2019). Postsurgical analgesia was performed with carprofen for 2 days.

One week after ihKA/ihNaCl injection, a subgroup of mice (*n*_NaCl_ = 5, *n*_KA_ = 6) was implanted with wire electrodes [platinum-iridium, teflon-insulated, ∅125 μm, World Precision Instruments (WPI), Sarasota, FL, USA] into the dentate gyrus (DG; AP = −2.0 mm, ML = ± 1.4 mm, DV = −1.9 mm) and the subiculum (AP = −3.0 mm, ML = ± 1.4 mm, DV = −1.4 mm) of one or both hemispheres (anesthesia see above, analgesia with buprenorphine+carprofen for 2 days). Jewelers’ screws above the frontal cortex served as ground/reference. A connector was permanently fixed to the skull with cyanoacrylate and dental cement. Freely moving mice were recorded (6−8 sessions, ∼2 h each) between 14 and 33 days after ihKA connected to a miniature preamplifier (Multi Channel Systems, Reutlingen, Germany). Signals were amplified (500-fold, band-pass filter 1 Hz–5 kHz) and digitized [sampling rate 10 kHz, Spike2 software; Cambridge Electronic Design (CED), Cambridge, UK]. Mice that died after surgery, with misplaced electrodes or with unsuccessful kainate injection (verified by *post-hoc* Nissl staining in frontal brain slices) were excluded (*n*_NaCl_ = 2, *n*_KA_ = 3).

### 2.3. Perfusion and slice preparation

Mice were transcardially perfused under deep anesthesia with 0.9% saline followed by paraformaldehyde [PFA, 4% in 0.1 M phosphate buffer (PB), pH 7.4]. Brains were post-fixed in PFA overnight (4°C), cryoprotected (30% sucrose) and frozen in isopentane. Horizontal sections (50 μm) were prepared on a cryostat and collected in PB for immunohistochemistry or in 2× saline sodium citrate (SSC) for fluorescence *in situ* hybridization (FISH).

### 2.4. FluoroJade C

FluoroJade C (FJC, Merck Millipore, Darmstadt, Germany) staining was used to monitor cell death 2 and 4 days after ihKA injection. Sections were mounted on gelatin-coated slides and dried overnight at RT, re-hydrated (1% NaOH in 100% Ethanol for 5 min; 70% Ethanol for 2 min, distilled water for 2 min), incubated in 0.06% potassium permanganate (10 min), rinsed and transferred in 0.0001% FJC solution (10 min, darkness), followed by washing, xylene immersion, and coverslipping with Hypermount.

### 2.5. Immunohistochemistry

After preincubation with 0.25% Triton X−100 and 10% normal horse/goat serum in 0.1 M PB (30 min, free-floating), sections were incubated overnight (4°C) with primary antibodies: guinea pig anti-NeuN (1:500; #266004, Synaptic Systems, Göttingen, Germany), rabbit anti-calretinin (CR; 1:500; #7699/4), rabbit anti-parvalbumin (PV; 1:1000; #PV27, all Swant, Burgdorf, Switzerland), and rabbit anti-NPY (1:1000; #ab30914, Abcam, Cambridge, UK). Secondary antibodies, marked with Cy2, Cy5 (both 1:200; Jackson ImmunoResearch, West Grove, PA, USA) or Alexa555 (1:500; Thermo Fisher Scientific, Waltham, MA, USA) were applied (2−3 h, RT). Sections were counterstained with 4’,6-diamidino-2-phenylindole (DAPI, 1:10,000). When combining FISH and immunohistochemistry, Triton X-100 was omitted. Sections were coverslipped with fluorescence mounting medium (Immu-Mount, Thermo Shandon Ltd., Schwerte, Germany or Dako, Hamburg, Germany).

### 2.6. Fluorescence *in situ* hybridization

Expression of *Gad67* mRNA was investigated by FISH with digoxigenin (DIG)-labeled cRNA probes. Probes were generated by *in vitro* transcription from appropriate plasmids (Kulik et al., 2003). Sections were hybridized overnight at 45°C, followed by detection with an anti-DIG antibody and tyramide signal amplification as described (Marx et al., 2013; Tulke et al., 2019).

### 2.7. Microscopy and quantification

Sections were imaged with an epifluorescence microscope (AxioImager 2, Zen software, Zeiss, Göttingen, Germany; 10-fold Plan Apochromat objective, NA = 0.45; AxioCam 506). Cell counting was performed with ImageJ (version 1.52a; NIH) on all available brain sections from DV −1.2 to −3.2 mm relative to bregma. The region of interest (ROI, principal cell layers of the subiculum) was drawn according to a brain atlas (Franklin and Paxinos, 2008). Cells were counted manually using the cell counter plugin. Criteria for selection were: signal intensity clearly distinguishable from background, cell shape and presence of a DAPI-stained nucleus. Blinding to the treatment was impossible due to KA-induced alterations in the hippocampus. To compare control and KA-injected mice along the DV axis, sections were assigned to the following subgroups: dorsal to the injection site level (−1.2 to −1.8 mm ventral to bregma–usually three sections/mouse), approximately at the injection site (−1.9 to −2.1 mm–2 sections/mouse) and ventral (−2.2 to maximally −3.2 mm – 4−6 sections/mouse). Cell density was calculated for the ROI (cells/mm^2^) and averaged within subgroups per animal. Values for the ipsi- and contralateral subiculum of NaCl-injected mice were grouped since we did not observe any differences. FluoroJade C-positive cells were detected in sections at the injection site as regional maxima in an intensity landscape using the image processing toolbox in Matlab (R2020b, The MathWorks Inc., Natick, MA, USA). A rectangular window spanning from the alveus to the molecular layer of 200 μm width was used as region of interest and divided into 20 equally sized bins to normalize for differences in width of the subiculum between individual sections. Counts for every bin were averaged across mice.

### 2.8. Statistics

Statistical analysis was performed with GraphPad Prism (version 9.3.1, GraphPad Software, Boston, MA, USA). After confirmation of normality with a Shapiro-Wilk test, a one-way analysis of variance (ANOVA) followed by Tukey’s multiple comparisons test was performed, otherwise a Kruskal–Wallis test was followed by Dunn’s multiple comparisons test.

## 3. Results

### 3.1. Epileptic activity occurs in the dentate gyrus and in the subiculum

**FIGURE 1:**
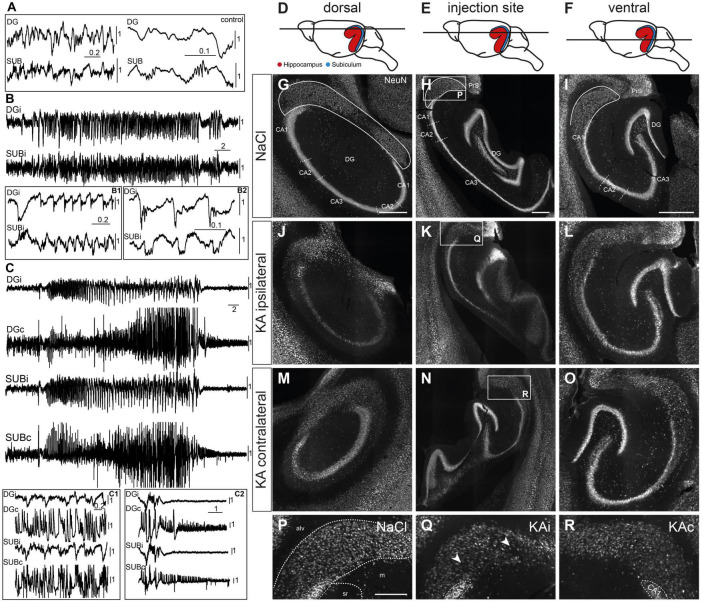
Epileptiform activity and neuronal loss in the subiculum. **(A)** Local field potential recording of a control mouse during awake immobility shows irregular activity in the dentate gyrus (DG) and subiculum (SUB) with sharp-wave-ripple complexes in the subiculum (enlarged traces at right side). **(B)** Kainate (KA)-injected mouse. Subclinical epileptic activity (during immobility, without behavioral correlate) involved the ipsilateral DG (DGi) and subiculum (SUBi). Epileptiform populations spikes are enlarged in panels **(B1,B2)**, panel **(B2)** also shows high frequency oscillations superimposed on population spikes in the DG and the subiculum. **(C)** Generalized convulsive seizure recorded in the DG and subiculum of both hemispheres. **(C1)** Cutout depicting large amplitudes on the contralateral side. Post-ictal depression **(C2)** starts earlier on the ipsilateral than on the contralateral side. Scale bars in panels **(A–C)**: horizontal–time in s, vertical–voltage in mV. Individually scaling of channels for visibility of characteristic patterns. **(D–F)** Schematic drawings represent the three investigated positions **(D)** dorsal, **(E)** injection site, **(F)** ventral along the dorso-ventral (DV) axis. Note that the subiculum (blue) is partly hidden behind the hippocampus (red) in the side view of the mouse brain. **(G–R)** NeuN immunostaining. Horizontal sections of the hippocampal formation from NaCl-injected control mice **(G–I)** were compared to 21 days after KA **(J–R)**. The position of the subiculum is encircled in panels **(G–I)**. **(J)** Ipsilateral side, dorsal position. At 21 days after KA the characteristic extensive cell loss in the CA regions confirms successful KA injection. Cell density in the subiculum is reduced. **(K)** Ipsilateral side, level of the injection site. Cell loss in the CA region and subiculum together with dispersion of the granule cell layer is visible. **(L)** Ipsilateral side, ventral position. The hippocampus and subiculum appear comparable to control. **(M–O)** Pattern of NeuN^+^ cells in the contralateral hippocampus and subiculum is comparable to controls at all DV levels. **(P–R)** Cutouts [white box in panels **(H,K,N)**]: subiculum at the level of the injection site. The borders of the subicular pyramidal cell layer toward the alveus (alv) and the molecular layer (m) are delineated as dashed lines in panel **(P)** according the brain atlas (Franklin and Paxinos, 2008). **(P)** Dense arrangement of neurons in the subiculum after NaCl injection. **(Q)** Decreased neuronal density in the ipsilateral subiculum after KA injection. Arrows mark positions of reduced cell density in the proximal subiculum close to CA1 and in the distal subiculum close to the border to the presubiculum. **(R)** The contralateral subiculum after KA injection is comparable to control, but NeuN staining is weaker in some mice. Scale bars: **(G–O)** 500 μm, **(P–R)** 250 μm. alv, alveus; CA, *cornu ammonis*; DG, dentate gyrus; m, molecular layer; PrS, presubiculum; sr, *stratum radiatum*.

In control mice (*n*_NaCl_ = 3), local field potential (LFP) patterns during immobility consisted of irregular activity and occasional dentate spikes in the DG and sharp-wave ripples (SWR) in the subiculum (Figure 1A). In ihKA mice (*n*_*KA*_ = 3) we recorded recurrent epileptic activity in the DG, as described previously (Häussler et al., 2012; Tulke et al., 2019), but also in the subiculum (Figure 1B). Fast ripples superimposed on large population spikes were evident in the DG (Figure 1B1) and in the subiculum where they resembled SWRs (Figure 1B2). In 2/3 mice generalized convulsive seizures occurred during the recording sessions which involved the ipsilateral DG and subiculum, and, in addition, the contralateral side where large epileptic population spikes were measurable (Figures 1C, C1). In agreement with previous observations (Lisgaras and Scharfman, 2022), it was impossible to determine the exact onset site whereas the post-ictal depression phase started with a delay on the contralateral side (Figure 1C2).

### 3.2. Position- and layer-specific neuronal loss in the subiculum

To determine whether the subiculum is structurally altered in chronic MTLE, we performed immunohistochemistry for NeuN at 21 days after ihKA (Figure 1, *n*_NaCl_ = 7 mice, *n*_*KA*_ = 7 mice). We will refer to the part of the subiculum adjoining CA1 as proximal and the area adjacent to the presubiculum as distal and regard the pyramidal cell layer along its radial axis using the alveus and the cell-poor molecular layer as borders, according to the brain atlas (Franklin and Paxinos, 2008). We compared three levels along the DV axis in horizontal sections: dorsal to the injection site (Figure 1D), around the injection site (Figure 1E) and ventral to the injection site (Figure 1F).

Extensive cell loss in areas CA1 and CA3 of the hippocampus at the dorsal and the injection site level, accompanied by salient granule cell dispersion at the level of the injection site confirmed the successful KA injection (Figures 1J, K), as described previously (Suzuki et al., 1995; Heinrich et al., 2006; Marx et al., 2013). Regarding the dorsal level, a reduced density of NeuN^+^ cells was visible in proximal and distal parts of the subiculum (Figure 1J) in comparison to controls (Figure 1G). At the level of the injection site NeuN^+^ cells were also thinned out in the proximal and distal ipsilateral subiculum (Figures 1H, P, K, Q), mainly in the lower half adjacent to the molecular layer (Figure 1Q). Moreover, many of the remaining neurons in the subiculum in both hemispheres displayed weaker NeuN staining than controls (Figures 1P-R). The latter was also visible in the ventral subiculum, yet without any clear sign of cell loss (Figure 1L) compared to control (Figure 1I). In the contralateral subiculum neuronal density was comparable to controls at all levels (Figures 1M-O, R).

**FIGURE 2:**
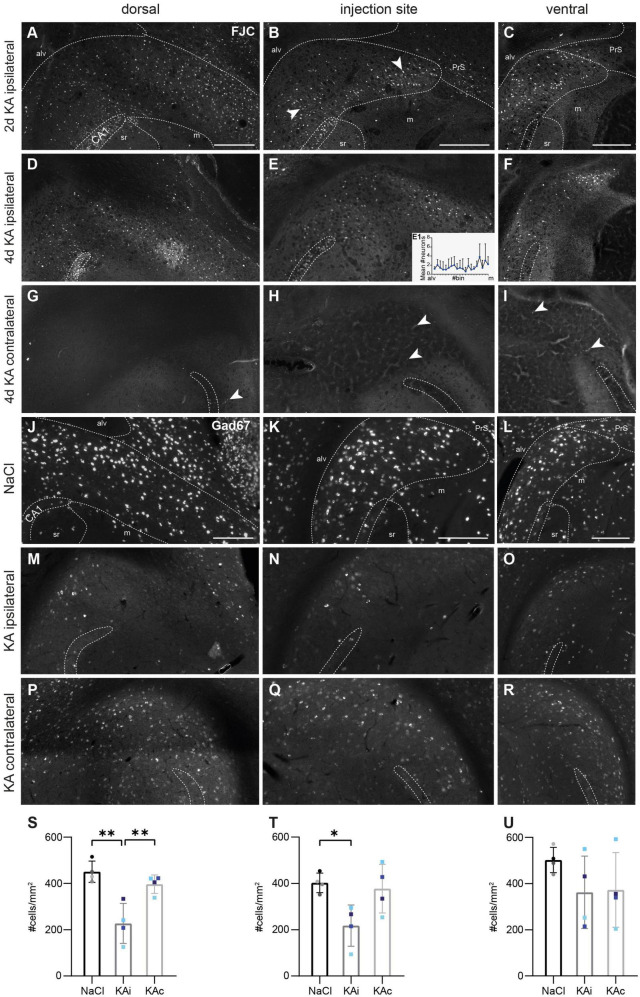
Degenerating neurons in the ipsilateral subiculum early after kainate (KA) injection and strong loss of GABAergic interneurons in the chronic stage. **(A–I)** Representative images of FluoroJade C (FJC)-stained degenerating neurons at 2 days **(A–C)** and 4 days after KA injection **(D–I)** along the DV axis. The localization of the CA1 pyramidal cell layer is indicated. The borders of the subicular pyramidal cell layer toward the alveus (alv) and the molecular layer (m) are delineated as dashed lines according the brain atlas (Franklin and Paxinos, 2008). **(A)** At 2 days after KA, FJC^+^ cells are distributed throughout the pyramidal cell layer of the dorsal ipsilateral subiculum. **(B)** At the level of the injection site, FJC-stained neurons are mainly located in the part of the pyramidal cell layer adjacent to the molecular layer. Degenerating neurons are densely placed in the proximal subiculum close to CA1 and distally, close to the presubiculum (arrowheads). **(C)** Similar pattern in the ventral subiculum. **(D–F)** At 4 days after KA, the distribution of FJC^+^ cells is similar but less dense at all DV positions. Mean cell density (mean + standard deviation, *n* = 4 mice) across the pyramidal cell layer (from alveus to molecular layer). Small differences in the transverse width of the subiculum are normalized by dividing the extent from alveus to molecular layer into 20 bins and averaging across bins. **(G–I)** In the contralateral subiculum, only a few neurons (arrowheads) were FJC^+^. **(J–R)** Representative horizontal sections of a fluorescence *in situ* hybridization (FISH) for *Gad67* mRNA in a control **(J–L)** and an epileptic **(M–R)** mouse 21 days after injection. **(J)** Dorsal subiculum and **(K)** level of the injection site after NaCl injection. GABAergic interneurons are equally distributed throughout the pyramidal cell layer of the proximal and distal subiculum. **(L)** Ventrally, the density of GABAergic interneurons is slightly higher. **(M,N)** Ipsilateral subiculum 21 days after KA injection, dorsal position **(M)** and injection site **(N)**. The density of *Gad67* mRNA expressing cells is strongly decreased. **(O)** At the ventral position *Gad67* mRNA-expressing cells are better preserved. **(P–R)** Contralateral subiculum 21 days after KA injection. The density of *Gad67* mRNA expressing neurons is comparable to control at all DV levels but *Gad67* mRNA labeling is weaker. **(S–U)** Quantification of *Gad67* mRNA-expressing cells in the subiculum of NaCl-injected mice (ipsi- and contralateral side combined), ipsi- and contralateral subiculum of KA-injected mice 21 days after injection. Individual mice are color-coded, bars show means (cells/mm^2^) with standard deviation (SD). **(S)** In the dorsal ipsilateral subiculum *Gad67* mRNA-expressing cells are significantly reduced compared to controls (one-way ANOVA: *p* = 0.002; Tukey’s multiple comparisons test: NaCl-KAi *p* = 0.002, KAi-KAc *p* = 0.009). **(T)** At the level of the injection site the density of *Gad67* mRNA-expressing cells in the ipsilateral subiculum is significantly reduced compared to control (one-way ANOVA: *p* = 0.024; Tukey’s multiple comparisons test: NaCl-KAi *p* = 0.029). **(U)** At the ventral position, density of GABAergic interneurons varies strongly across epileptic mice, means are not different (one-way ANOVA *p* = 0.305). Scale bars: 250 μm. *p < 0.05, **p < 0.01. alv, alveus; CA1, *cornu ammonis* 1; m, molecular layer; PrS, presubiculum; sr, *stratum radiatum*.

To determine whether the reduced density was due to SE-induced neurodegeneration, we performed FJC staining at 2 and 4 days after ihKA injection. At 2 days, degenerating neurons were densely arranged in ipsilateral CA1 (Figures 2A, B) and all over the ipsilateral hippocampus (data not shown), as described previously (Marx et al., 2013). In the subiculum, FJC^+^ cells were abundant throughout the transverse axis of the pyramidal cell layer at the dorsal position, whereas at the injection site and the ventral position, they tended to be denser in the lower half located adjacent to the molecular layer than close to the alveus (Figures 2A-C, *n*_KA_ = 2 mice). At 4 days after ihKA, FJC^+^ cells showed a similar distribution, but with overall lower density (Figures 2D-F, mean spatially resolved cell density at the level of the injection site in Figure 2E1, *n*_KA_ = 4 mice). On the contralateral side there were only very few FJC^+^ cells both in the hippocampus and the subiculum at all levels for both time points (Figures 2G-I, 2 days not shown).

Together, early cell loss and reduced neuronal density in the chronic stage confirm ihKA-induced structural changes in the ipsilateral subiculum, yet much less prominent than in the hippocampus. Next, we focused on selected IN populations.

### 3.3. Loss of GABAergic interneurons in the subiculum

In controls, GABAergic INs, as displayed by FISH for *Gad67* mRNA, were densely arranged throughout the pyramidal cell layer of the proximal and distal subiculum (Figure 2J-L, *n*_NaCl_ = 4 mice). Quantitative analysis did not reveal any major differences between the dorsal subiculum and the level of the injection site (Figures 2J-L, S, T) but in the ventral subiculum, the density of *Gad67* expressing cells was slightly higher (Figure 2U).

At 21 days after ihKA (*n*_KA_ = 4 mice), *Gad67* mRNA^+^ INs were strongly diminished throughout the ipsilateral dorsal subiculum (Figure 2M) and at the level of the injection site (Figure 2N), and in some mice they still appeared less dense further ventrally (Figure 2O). In addition, the *Gad67* FISH was less intense indicating reduced mRNA levels per cell. In the contralateral subiculum, the density of *Gad67*^+^ cells was comparable to control at all levels but the staining intensity was also lower (Figures 2P-R). Quantification in the ipsilateral subiculum revealed the loss of ∼50% of *Gad67*^+^ INs at the dorsal position and at the level of the injection site (Figures 2S, T; dorsal: one-way ANOVA *p* = 0.002, Tukey’s multiple comparison test NaCl-KAi *p* = 0.002, NaCl-KAc *p* = 0.453, KAi-KAc *p* = 0.008; injection site: one-way ANOVA *p* = 0.024, Tukey’s multiple comparison test NaCl-KAi *p* = 0.029, NaCl-KAc *p* = 0.906, KAi-KAc *p* = 0.057). Ventrally, mean values did not differ from controls but individuals ranged from a strong loss to normal density (Figure 2U; one-way ANOVA *p* = 0.305).

### 3.4. Loss of parvalbumin- and calretinin-positive interneurons in the ipsilateral subiculum

**FIGURE 3:**
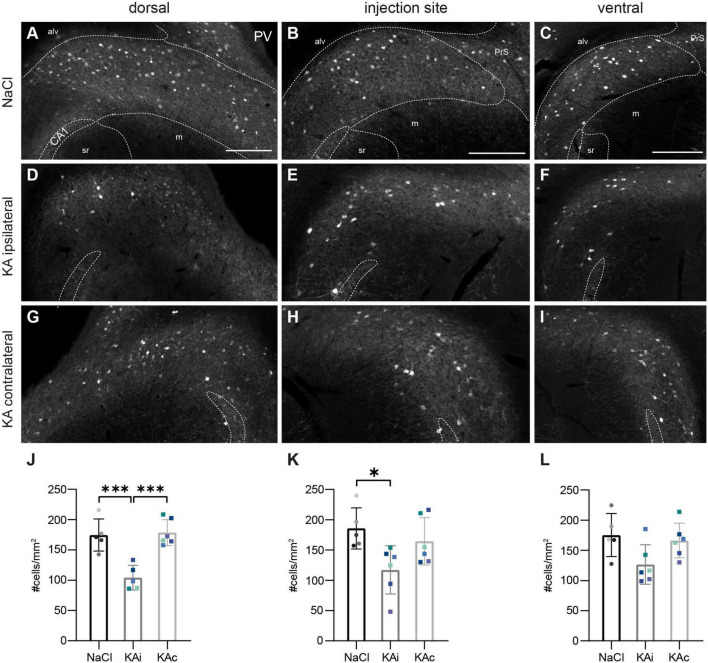
Parvalbumin (PV)-positive interneurons are reduced in the ipsilateral subiculum 21 days after kainate (KA). Representative sections immunostained for PV in control mice **(A–C)** and 21 days after KA injection **(D–I)**. The localization of the CA1 pyramidal cell layer is indicated. The borders of the subicular pyramidal cell layer toward the alveus (alv) and the molecular layer (m) are delineated as dashed lines according the brain atlas (Franklin and Paxinos, 2008). **(A)** Dorsal subiculum after NaCl injection. PV^+^ cells are evenly distributed throughout the pyramidal cell layer of the proximal and distal subiculum and PV^+^ fibers form a dense plexus. **(B,C)** Similar pattern at the level of the injection site **(B)** and further ventral **(C)**. **(D)** Ipsilateral subiculum 21 days after KA, dorsal position. PV^+^ somata and PV^+^ fibers are strongly diminished, mainly in the part of the pyramidal cell layer adjacent to the molecular layer. **(E)** Similar pattern for the level of the injection site and further ventral **(F)**. **(G–I)** Contralateral subiculum 21 days after KA. The density of PV^+^ interneurons is comparable to controls. **(J–L)** Quantification of PV^+^ cells in the subiculum of NaCl-injected mice, ipsi- and contralateral subiculum of KA-injected mice 21 days after injection. Individual mice are color-coded, bars show means (cells/mm^2^) with standard deviation (SD). **(J)** Reduced density of PV^+^ cells in the ipsilateral (KAi) but not contralateral dorsal subiculum (KAc) (one-way ANOVA: *p* = 0.0002, Tukey’s multiple comparison test NaCl–KAi: *p* = 0.0008; KAi–KAc: *p* = 0.0004). **(K)** Reduction of PV^+^ cell density in the ipsilateral subiculum at the injection site (one-way ANOVA: *p* = 0.026, Tukey’s multiple comparison test NaCl–KAi: *p* = 0.025). **(L)** The effect on KA on PV^+^ interneurons is weaker in the ventral subiculum (one-way ANOVA: *p* = 0.051). Scale bars: 250 μm. ***p < 0.001, *p < 0.05. alv, alveus, CA1, *cornu ammonis* 1, m, molecular layer, PrS presubiculum, sr *stratum radiatum*.

In control mice (*n*_NaCl_ = 5), INs expressing the Ca^2+^-binding protein PV were scattered throughout the pyramidal cell layer of the subiculum with their fibers building a honeycomb-like pattern reaching into the molecular layer (Figures 3A-C). Their density was comparable at all DV positions (Figures 3J-L).

At 21 days after ihKA (*n*_KA_ = 6 mice), we observed a reduction of PV^+^ INs at all DV positions of the ipsilateral subiculum (Figures 3D-F), but most pronounced dorsally (Figure 3D) with PV^+^ cells located close to the molecular layer being slightly more affected than those close to the alveus (Figures 3D-F). Quantitative analysis confirmed a significant reduction of PV^+^ INs in the dorsal ipsilateral subiculum by ∼40% (Figure 3J; one-way ANOVA *p* = 0.0002, Tukey’s multiple comparison test NaCl-KAi *p* = 0.0008, NaCl-KAc *p* = 0.955, KAi-KAc *p* = 0.0004) and at the injection site by ∼36% (Figure 3K; one-way ANOVA *p* = 0.026, Tukey’s multiple comparison test NaCl-KAi *p* = 0.025, NaCl-KAc *p* = 0.634, KAi-KAc *p* = 0.113). The reduction of mean density in the ventral subiculum did not reach significance (Figure 3L; one-way ANOVA *p* = 0.051). On the contralateral side, the distribution and density of PV^+^ cells were comparable to control at all positions (Figures 3G-L).

**FIGURE 4:**
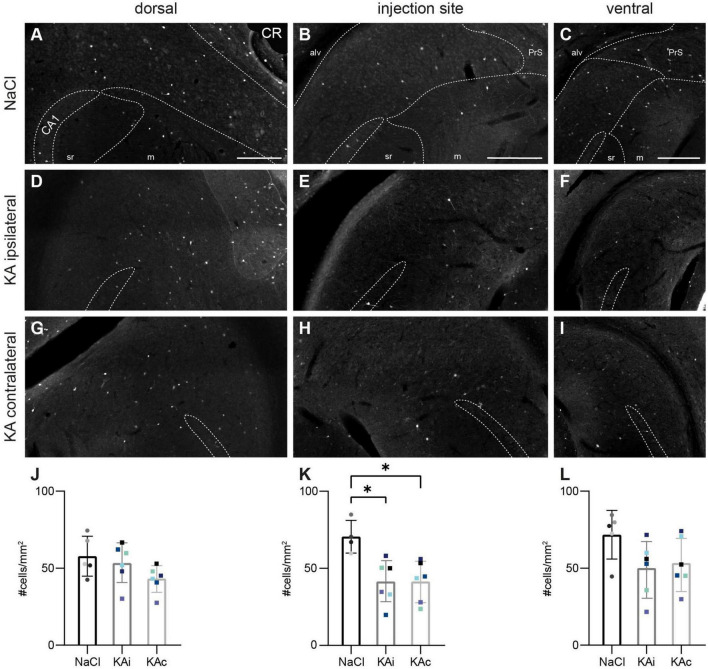
Calretinin (CR)-expressing neurons are reduced 21 days after kainate (KA) injection. Representative sections immunostained for CR in control mice **(A–C)** and 21 days after KA injection **(D–I)**. The localization of the CA1 pyramidal cell layer is indicated. The borders of the subicular pyramidal cell layer toward the alveus (alv) and the molecular layer (m) are delineated as dashed lines according the brain atlas (Franklin and Paxinos, 2008). **(A–C)** In the subiculum of control animals CR^+^ neurons are sparsely scattered throughout the pyramidal cell layer at the dorsal position **(A)**, the injection site **(B)** and more ventral **(C)**. **(D)** At 21 days after KA, the density and distribution of CR^+^ cells is comparable to controls in the dorsal ipsilateral subiculum. **(E)** At the injection site, CR^+^ cells are reduced throughout the pyramidal cell layer. **(F)** At the ventral position, the density of CR^+^ cells is reduced in some, but not all mice. **(G)** In the dorsal contralateral subiculum, the density of CR^+^ cells is comparable to control. **(H)** At the level of the injection site, CR^+^ cells are reduced also on the contralateral side. **(I)** Further ventral, CR immunolabeling is comparable to the ipsilateral side with reduced density in some mice. **(J–L)** Quantification of CR^+^ cells in the subiculum of NaCl-injected mice, ipsi- and contralateral subiculum of KA-injected mice 21 days after injection. Individual mice are color-coded, bars show means (cells/mm^2^) with standard deviation (SD). **(J)** At the dorsal position the means of the three tested groups do not differ (one-way ANOVA: *p* = 0.138). **(K)** At the level of the injection site, the density of CR^+^ cells is reduced at the ipsilateral and contralateral side compared to control (one-way ANOVA: *p* = 0.007; Tukey’s multiple comparisons test: NaCl-KAi *p* = 0.010, NaCl-KAc *p* = 0.012). **(L)** No significant differences at the ventral position (one-way ANOVA: *p* = 0.123). Scale bars: 250 μm. *p < 0.05. alv, alveus; CA1, *cornu ammonis* 1; m, molecular layer; PrS, presubiculum; sr, *stratum radiatum*.

Next, we analyzed the distribution of cells expressing the Ca^2+^-binding protein CR. These INs were much sparser than PV^+^ INs and scattered throughout the pyramidal cell layer of all DV levels in controls (*n*_NaCl_ = 5 mice, Figures 4A-C).

At 21 days after ihKA we did not observe any major changes in the dorsal subiculum of the ipsi- or contralateral hemisphere, respectively, (*n*_KA_ = 6 mice; Figures 4D, G, J; one-way ANOVA *p* = 0.138). In contrast, at the level of the injection site the density of CR-expressing cells was reduced throughout the pyramidal cell layer of the ipsilateral (Figures 4E, H) and contralateral subiculum (Figures 4E, H, K, one-way ANOVA *p* = 0.007, Tukey’s multiple comparison test NaCl-KAi *p* = 0.010, NaCl-KAc *p* = 0.012, KAi-KAc *p* = 0.996). In ventral parts, CR^+^ cells were reduced in some mice in both hemispheres (Figures 4F, I), whereas others showed normal distribution of CR^+^ cells. This is reflected in high variability in the quantification, but no significant differences (Figure 4L, one-way ANOVA *p* = 0.123).

### 3.5. Reduction of neuropeptide Y-expressing interneurons but compensatory NPY upregulation

**FIGURE 5:**
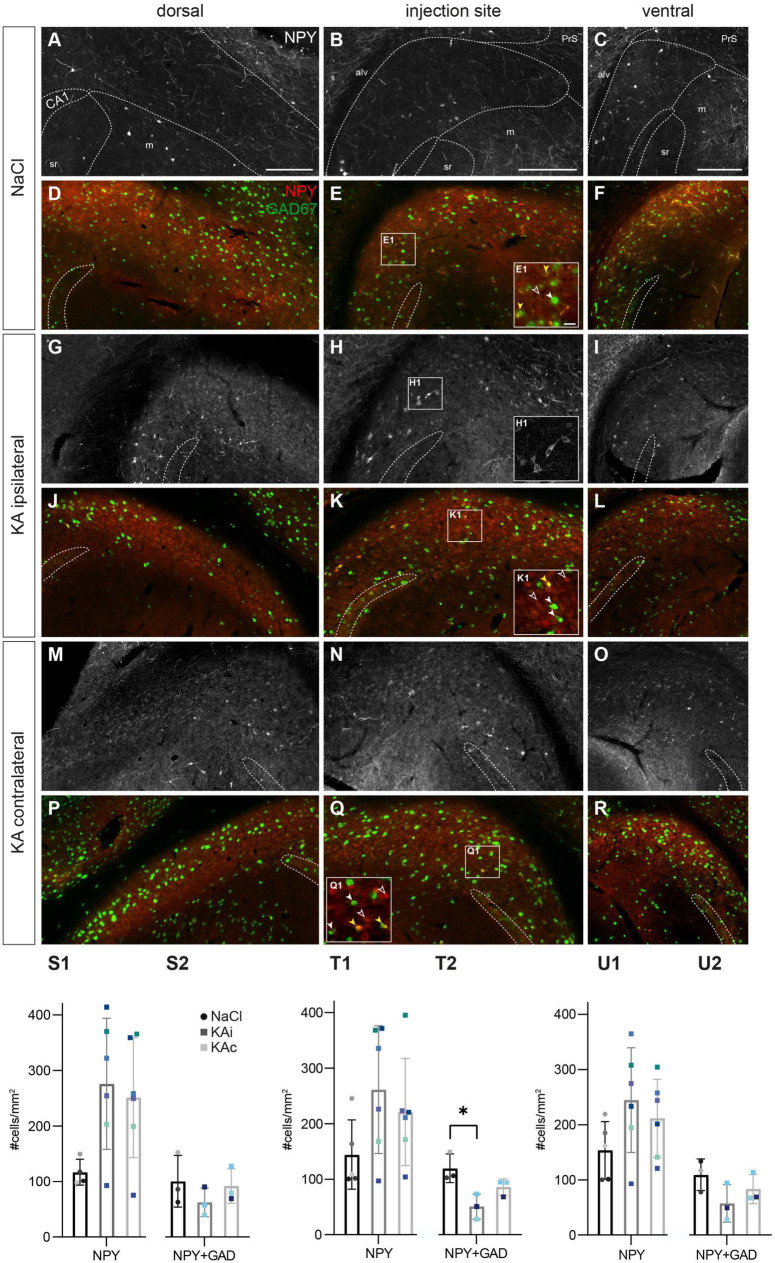
Kainate (KA) induces an upregulation of neuropeptide Y (NPY), but loss of NPY interneurons. Representative images depicting the subiculum of control **(A–F)** and KA-injected mice **(G–R)**. Immunolabeling of NPY^+^ cells [red, **(A–C,G–I,M–O)**] and counterstaining for co-expression of *Gad67* mRNA using fluorescence *in situ* hybridization [green, **(D–F,J–L,P–R)**]. The localization of the CA1 pyramidal cell layer is indicated. The borders of the subicular pyramidal cell layer toward the alveus (alv) and the molecular layer (m) are delineated as dashed lines according the brain atlas (Franklin and Paxinos, 2008). **(A)** NaCl-injected control, dorsal subiculum. NPY^+^ cells and their processes are located throughout the pyramidal cell layer more of the proximal than the distal subiculum. **(B)** Similar distribution of the injection site and **(C)** the ventral subiculum. **(D–F)** High density of *Gad67* mRNA^+^ neurons (green) at all positions along the DV axis in control mice. A subset of these cells is NPY^+^ (overlay in yellow), whereas a few NPY^+^ cells are negative for *Gad67* mRNA (red only). **(E1)** Enlarged cutout marked in panel **(E)** with double-positive cells (yellow overlay, yellow arrowheads mark examples), *Gad67*^+^ but NPY^–^ neurons (green, closed white arrowheads) and *Gad67*^–^ but NPY^+^ cells (red, open arrowheads). **(G–I)** At 21 days after KA, NPY expression is strongly increased at all DV levels of the ipsilateral subiculum in cells of various shapes and sizes [examples are enlarged in panel **(H1)**]. **(J–L)** The density of *Gad67* mRNA^+^ cells is decreased in the ipsilateral subiculum after KA. Only a subset is NPY-positive. **(K1)** Enlarged cutout marked in panel **(K)** shows one double-positive cell (yellow arrowhead), a reduced density of *Gad67*^+^/NPY^–^ neurons (closed white arrowheads) and increased density of *Gad67*^–^/NPY^+^ cells (red, open arrowheads). **(M–O)** In the contralateral subiculum of KA-injected mice, NPY expression is upregulated at all positions along the DV axis. **(P–R)** The distribution of *Gad67* mRNA^+^ neurons and of double-labeled cells is comparable to the control condition, but the density of NPY^+^ cells is increased. **(Q1)** Enlarged cutout marked in panel **(Q)**. **(S1–U1)** Quantification of NPY^+^ cells in controls and at 21 days after KA. Individual values (color-coded for mice), means and standard deviation (SD) are displayed. Individuals vary largely and differences are not significant. **(S2–U2)** Quantification of the density of NPY^+^/Gad67^+^ double-labeled cells (i.e., NPY^+^ GABAergic interneurons) in the subiculum. A trend for lower values in the ipsilateral subiculum after KA is visible for all DV positions, but reaches significance only at the injection site [**(T2)**: Kruskal–Wallis test: *p* = 0.011, Dunn’s multiple comparison test: NaCl-KAi *p* = 0.034]. Scale bars: **(A–R)** 250 μm, **(F1)** 50 μm. *p < 0.05. alv, alveus; CA1, *cornu ammonis* 1; m, molecular layer; PrS, presubiculum; sr, *stratum radiatum*.

In control mice (*n*_NaCl_ = 5), NPY^+^ neurons were sparsely distributed throughout the pyramidal cell layer mainly of the proximal and to a much lesser extent the distal subiculum (Figure 5A) with increasing mean density from dorsal to ventral (Figures 5S1-U1). Their intense arborizations extended into the molecular layer (Figures 5B, C).

At 21 days after ihKA (*n*_KA_ = 6 mice), NPY staining was strongly increased in most mice reflected by a much higher density of NPY^+^ somata and axonal processes at all DV and proximo-distal levels of the ipsilateral (Figures 5G-I) and contralateral subiculum (Figures 5M-O). Shapes, sizes and the staining intensity of NPY^+^ cells appeared very diverse after KA injection (Figure 5H1). Quantitative analysis revealed a high variability in the density of NPY^+^ cells ranging from unchanged density to a fourfold increase along the entire DV axis of the ipsilateral subiculum, but the means were not significantly different (Figures 5S1-U1; one-way ANOVA dorsal *p* = 0.068, injection site *p* = 0.163, ventral *p* = 0.174). A similar variability with strongly elevated density in some but not all mice was also observable in the contralateral subiculum.

Is there an increase of NPY-expressing INs in the subiculum? In our previous study regarding the hippocampus proper we found a transient upregulation in excitatory cells alongside a decreased number of NPY^+^ INs (Marx et al., 2013). To test this in the subiculum, we combined NPY immunohistochemistry with FISH for *Gad67* mRNA in a subset of samples. In controls, the majority of NPY^+^ neurons were also *Gad67*-positive (*n*_NaCl_ = 3 mice; Figures 5D-F, E1), but a small number of NPY^+^/*Gad67*^–^ cells (Figures 5E, E1) explains the overall smaller numbers of double-stained cells compared to NPY alone in the quantification of controls (Figures 5S2-U2).

In contrast, at 21 days after ihKA, a subset of the NPY^+^ cells co-expressed *Gad67* mRNA, whereas most were negative (NPY^+^/*Gad67^–^)*, indicating the upregulation of NPY in non-GABAergic cells of both hemispheres (*n*_KA_ = 3 mice, Figures 5J-L, P-R, K1, Q1). In the contralateral subiculum, the density and distribution of NPY^+^/*Gad67*^+^ double-positive neurons were comparable to controls (Figures 5S2-U2). In the ipsilateral subiculum, quantification of NPY^+^/*Gad67*^+^ cells revealed a tendency toward lower numbers of double-labeled cells which was significant at the level of the injection site (Figures 5S2-U2; dorsal one-way ANOVA *p* = 0.441, injection site Kruskal-Wallis test *p* = 0.011, Dunn’s multiple comparison test NaCl-KAi *p* = 0.034, NaCl-KAc *p* = 0.408, KAi-KAc *p* = 0.890, ventral one-way ANOVA *p* = 0.184). Together our data show a loss of NPY^+^ INs and NPY upregulation in the subiculum in non-GABAergic cells.

## 4. Discussion

Our study provides a comprehensive insight into the pathological changes of the subiculum in a focal MTLE model with unilateral hippocampal sclerosis. Degenerating neurons in the ipsilateral subiculum were detected already 2 days after ihKA and neuronal density was persistently lower in chronic epilepsy. Specifically, we found the loss of GABAergic cells, mainly of PV^+^ INs and to a lesser extent of CR^+^ and NPY^+^ INs with location-dependent patterns for the individual populations. In contrast, NPY was upregulated in non-GABAergic cells throughout the subiculum of both hemispheres. Together, our data show that in the ihKA mouse model, the subiculum is involved in epileptic activity and undergoes epilepsy-associated histological changes of similar nature but much less pronounced than in the dorsal ipsilateral hippocampus.

The degeneration of neurons early after ihKA injection is in agreement with the hypothesis that cell death in the subiculum contributes to epileptogenesis in the network beyond the hippocampus. Neuronal degeneration was nearly equally pronounced at the dorsal position and at the level of the injection site. With increasing distance from the injection site, the subiculum was better preserved in agreement with our previous studies investigating the hippocampus (Häussler et al., 2012; Marx et al., 2013; Janz et al., 2017b). Interestingly, the part of the pyramidal cell layer closer to the molecular layer was more affected than that close to the alveus, in particular for GAD- and PV-positive INs, suggesting a position-dependent differential vulnerability. Indeed, expression of molecular markers, intrinsic firing patterns and in- and outward connectivity of principal cells in the subiculum differ along the proximo-distal as well as the transverse axis (Cembrowski et al., 2018a,b), which might render them differentially vulnerable. A similar position-dependent diversity of neuronal properties among IN populations is also conceivable. Otherwise, different interaction patterns with the individual principal cell populations in the individual layers might also lead to selective vulnerability. Finally, external inputs, e.g., those expressing the vesicular glutamate transporter 2 (vGlut2) and Zink transporter 3 (ZnT3) are not equally distributed along the DV and transversal axes of the subiculum (Ishihara and Fukuda, 2016) and might lead to different degrees of overexcitation during *status epilepticus* and in chronic epilepsy.

**FIGURE 6:**
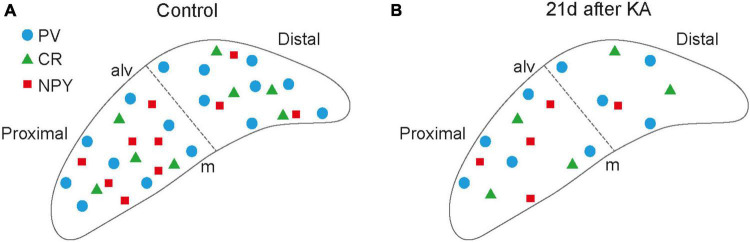
Schematic summary figure. Schematic illustration of placement of subicular interneurons under control **(A)** versus epileptic conditions 21 days after kainate (KA) **(B)** in the pyramidal cell layer of the subiculum at the level of the injection site. Individual interneuron types are given in the legend. NPY^+^ neurons in the scheme only correspond to NPY-expressing interneurons (i.e., *Gad67* mRNA-positive). alv, alveus; m, molecular layer.

In comparison to the hippocampus, the distribution of IN populations in the subiculum is less well described. We therefore chose *Gad67* as a general IN marker and selected three markers of specific populations to first map their distribution in controls under our experimental conditions (Figure 6). INs expressing PV were the largest fraction with somata throughout the pyramidal cell layer and processes extending into the molecular layer. We found PV^+^ INs distributed nearly equally in the proximal and distal subiculum, which is in accordance with previous work (Fujise et al., 1995). CR^+^ cells were much sparser and scattered throughout the pyramidal cell layer of the proximal and distal subiculum. Cells expressing NPY, of which most were GABAergic in controls, showed a gradient from dorsal to ventral and were more numerous in the proximal than in the distal subiculum. To complement the analysis of IN populations expressing specific Ca^2+^-binding proteins, we initially also performed immunostaining for the calbindin, which is not only a marker for INs, but also for subicular principal cell populations (Fujise et al., 1995; Ishihara and Fukuda, 2016). However, the staining patterns under epileptic conditions were too diffuse for analysis and therefore discarded. For the present study, we did not map INs expressing SOM, cholecystokinin (CCK) or vasoactive intestinal peptide (VIP), which based on the density of GAD^+^ cells might comprise around 25−30% of INs and will be of relevance in future studies.

In ihKA mice, PV^+^ INs were lost in the dorsal subiculum and at the injection site (40 and 36% reduction, respectively), which is similar or eventually slightly less than in systemic rat and mouse MTLE models [30−60% loss, (Knopp et al., 2005; He et al., 2010; Drexel et al., 2011)]. In contrast, at the ventral position, the density of PV^+^ INs varied between normal and reduced density which is in agreement with observations we made earlier in the hippocampus (Marx et al., 2013) and might depend on the intensity of SE and the DV extent of hippocampal sclerosis. This could eventually serve as an explanation why findings on PV^+^ INs in the subiculum of humans are controversial (Arellano et al., 2004; Andrioli et al., 2007), as this might depend on the degree of sclerosis and the localization of the analyzed tissue along the hippocampal length axis. In accordance with the unilateral injection and unchanged numbers of *Gad67* mRNA-expressing cells, there was no major loss of PV^+^ INs in the contralateral subiculum, suggesting that generalized seizures that also affect the contralateral subiculum as shown by our intracranial recordings, do not lead to major IN loss. It is, thus, more likely that ipsilateral damage associated with direct cytotoxic effects of KA is detrimental to the ipsilateral PV^+^ cell population. For CR^+^ INs we found a reduction at the injection site whereas at the dorsal site their density was nearly normal, which differed from *Gad67*^+^ and PV^+^ populations. Generally, controversial data on CR exist for the human subiculum in MTLE ranging from a reduction (Maglóczky et al., 2000) to normal density (Blümcke et al., 1996), and high variability was also evident in systemic rat models (Knopp et al., 2008; Drexel et al., 2011). A solely local effect focused to the injection site is an unlikely explanation, as the reduction also affects ventral parts in some mice. Instead, the heterogeneous nature of CR-expressing neurons might play a role and require more elaborate investigation of subpopulation vulnerability with different double labeling approaches along the DV axis. Interestingly, the reduction also affected the contralateral subiculum despite no or only little detectable cell loss and no reduction of *Gad67*^+^ cells. This suggests that not necessarily a loss of CR^+^ cells, but also position- or neuron type-dependent downregulation of CR might play a role. To investigate this, fate mapping of those neurons in mouse lines with CR-dependent expression of Cre recombinase will be necessary to determine whether CR^+^ cells are lost or CR expression is downregulated, and to find out, whether this is associated with altered Ca^2+^ buffering capacity or might be compensated by upregulation of other Ca^2+^-binding proteins. Double-labeling for NPY and *Gad67* mRNA revealed that these INs were also vulnerable to the ihKA injection, albeit less than PV^+^ INs in agreement with observations in the hippocampus (Marx et al., 2013). As mentioned earlier, we did not analyze populations expressing SOM, VIP or CCK, but the strong loss of *Gad67* mRNA-expressing cells in the dorsal subiculum suggests also reductions for these populations.

How might the reduced IN density affect population activity in the subiculum? It has been shown that during SWRs in the LFP subicular pyramidal cells either increase or decrease their firing and that these divergent cell types correspond to bursting and regular firing neuron types, respectively (Böhm et al., 2015). Interestingly, regular firing cells receive stronger synaptic inhibition during SWRs, in particular from PV^+^ INs which themselves are strongly activated during SWRs. Given the reduction in PV^+^ (and other) INs after ihKA injection, a shift toward more cells in bursting mode and hence from physiological SWRs to pathologic epileptic bursts is conceivable. In addition, despite the loss of CA1 input to the subiculum, projections from CA2 persist (own unpublished observations) and inputs from the entorhinal cortex, which strongly target the subiculum (Steward, 1976; Cembrowski et al., 2018a) are also very likely to persist as we have shown it for the DG (Janz et al., 2017b). In coincidence with reduced feed-forward inhibition these excitatory projections might strongly activate the subiculum. Such high excitatory pressure might also cause compensatory mechanisms to restore network balance. Indeed, we observed the upregulation of NPY after ihKA, comparable to epileptic rats (Drexel et al., 2012a). Various *in vitro* and *in vivo* studies in animal models (Woldbye et al., 1996; Klapstein and Colmers, 1997), as well as in human tissue (Patrylo et al., 1999; Ledri et al., 2015) have shown NPY acting as endogenous anti-convulsant in the hippocampal network. The upregulation did not only affect the ipsilateral, but also the contralateral subiculum, which presents with no or only little cell loss. This indicates that whereas cell loss might be an early effect due to direct effects of KA or cell-intrinsic processes triggered by hyperexcitation during *status epilepticus*, compensatory processes might apply to protect neurons in both hemispheres from generalized seizures or to reduce overall excitability. Determining whether subicular NPY plays a role in seizure suppression in the subiculum will require experiments with local knock-out of NPY or its receptors.

In summary, our data point toward an important role of the subiculum in the epileptic network by undergoing a 50% loss of INs and compensatory processes. The fact that the subiculum shows changes similar in nature as the hippocampus, but less intense, might render it into a hub which is disinhibited enough, and on the other hand presents sufficient neuronal preservation to contribute to the generation and/or the propagation of epileptic activity from the hippocampus to other parts of the brain.

## Data availability statement

The raw data supporting the conclusions of this article will be made available by the authors, without undue reservation.

## Ethics statement

This animal study was reviewed and approved by the Regierungspräsidium Freiburg, Abteilung Landwirtschaft, Ländlicher Raum, Veterinär- und Lebensmittelwesen, Freiburg, Germany.

## Author contributions

UH and ST: conceptualization. JF, NB, HW, ST, and UH: experimental work. JF, NB, HW, and UH: data analysis. UH and CH: funding acquisition. NB and UH: visualization. JF, NB, and UH: writing—original draft preparation. CH, ST, and HW: writing—review and editing. All authors contributed to the article and approved the submitted version.
